# A large two-centre study in to rates of influenza and pneumococcal vaccination and infection burden in rheumatoid arthritis in the UK

**DOI:** 10.1186/s12891-016-1187-4

**Published:** 2016-08-04

**Authors:** Sujith Subesinghe, Andrew Ian Rutherford, Fowzia Ibrahim, Helen Harris, James Galloway

**Affiliations:** 1 Department of Rheumatology, King’s College Hospital, Denmark Hill, London, SE5 9RT England, UK; 2 Academic Department of Rheumatology, King’s College London, Weston Education Centre, Denmark Hill, London, SE5 9RJ England, UK; 3 NIHR Biomedical Research Centre, Guy’s Hospital, Great Maze Pond, London, SE1 9RT England, UK; 4 Fife Rheumatic Diseases Unit, Whytemans Brae Hospital, Kirkcaldy, Fife, KY1 2ND Scotland, UK

**Keywords:** Rheumatoid arthritis, Infection, Pneumococcal vaccines, Influenza vaccines

## Abstract

**Background:**

Infections are a common complication of RA with associated morbidity and mortality. The aetiology of increased risk is complex and multifactorial. Despite this, strategies to mitigate against risk of infection including vaccination are not always addressed in primary or secondary care with wide variation in practice from multiple small single centre audits. This study was a large two-centre survey of vaccine uptake in routine clinical practice and evaluated the relationship between vaccination and the burden of infection in RA patients.

**Methods:**

A patient questionnaire was devised and disseminated through postal, clinic and phone survey at 2 UK rheumatology centres, detailing past vaccination history, reasons for non-vaccination, and history of recent infection. In a subset of patients, primary care vaccination data were also obtained.

**Results:**

In total 929 patients responded to the survey. Over 85 % of patients were vaccinated against influenza, however only 44 % were vaccinated against pneumococcus. The vast majority of vaccination was undertaken in primary care. In the 12 months prior to the survey, 7.7 % of subjects recalled at least one episode of severe infection requiring admission, and nearly 40 % reported receiving at least one course of antibiotics.

**Conclusions:**

Infections are common in RA and Rheumatologists need to be adept at recognising at risk patients and managing them appropriately. Influenza vaccination uptake is good whilst pneumococcal vaccination rates are comparatively poor. Collaborative approaches between primary and secondary care are required to maximise vaccine uptake, which is safe and recommended in RA patients.

**Electronic supplementary material:**

The online version of this article (doi:10.1186/s12891-016-1187-4) contains supplementary material, which is available to authorized users.

## Background

Rheumatoid Arthritis (RA) is a chronic autoimmune inflammatory disorder that if untreated results in damage to joints with irreversible changes that confer increased morbidity and functional loss [1]. Patients with RA have an increased burden of infections [2]. A retrospective longitudinal cohort study by Doran et al. reported on 609 RA subjects with over 7700 patient years of cumulative follow up, during which the infection rate was 70–80 % higher in RA subjects than controls [3]. Subsequent cohort studies have reported similar findings [4, 5]. The increased infection risk in RA is due to an interaction between immunological dysfunction, exposure to potent immunosuppressive agents and inherent disease activity [6–9]. Approximately half of all serious infections in RA patients are attributable to a respiratory tract focus. National death registry data have confirmed an increased standardised mortality ratio (SMR) in this population (SMR males 1.8 [95 % Confidence Interval [CI] I 1.6, 2.0]; SMR females 2.1 [95 % CI 1.9, 2.3]) [10, 11]. Pneumococcus is the most frequently causative microbe in patients with pneumonia, responsible for approximately 50 % of cases [12, 13].

Rheumatologists need to be adept at managing infections as a consequence of therapies and taking opportunities to mitigate infection risk is a logical step. Vaccination is a key example. Guidance from the British Society of Rheumatology (BSR) and the Joint Committee on Vaccination and Immunisation (JCVI) recommend that patients with autoimmune rheumatic diseases should be offered a single pneumococcal vaccination (with a second ‘booster’ vaccination after 5 years in severely immunocompromised individuals) and receive seasonal influenza vaccination annually in accordance with national guidelines. Vaccination in RA patients is safe with no evidence to suggest worsening of disease post-administration, however vaccination uptake remains poor with published single-centre audits showing wide variation in practice (vaccination uptake between 22 and 100 %) [14–16].

## Methods

We undertook a clinical audit of vaccine uptake to determine the proportion of RA patients receiving seasonal influenza and pneumococcal vaccination, identify factors associated with vaccination uptake and explore the reasons for non-vaccination.

A cross sectional survey of vaccine uptake in RA patients was undertaken at 2 U.K. sites (King’s College Hospital, London (KCH) and Fife Rheumatic Diseases Unit, Scotland) between January and March 2014. A patient questionnaire was developed in collaboration with local and national rheumatologists (see Additional file 1). The questionnaire was reviewed by a patient focus group prior to dissemination via postal survey with additional data capture through clinic attendance and telephone survey. By completing the questionnaire, subjects were giving their consent to participate. Data collected included prior vaccination history measuring uptake against national standards. The vaccination history time interval covered by the survey was not specified and any history of vaccination was captured. In a subset of patients, vaccination history was confirmed by primary care providers. At KCH, hospital episode statistics (HES) submission data review alongside analysis of the electronic record with discharge summaries pertaining to admission was undertaken to validate patient reported data regarding hospitalised infection (as confirmed by ICD-10 coding).

Summary statistics were analysed using parametric and non-parametric tests as appropriate, logistic regression was used to explore the relationship between vaccination and infection as provided in the survey data. Subsequent analyses using data available from HES utilised survival modelling, using Cox proportional hazards. Adjustment for confounding variables was performed using an inverse probability of treatment weighting (IPTW) model. A logistic regression analysis was performed to calculate the probability of each patient being in the exposed or unexposed group based upon their clinical characteristics chosen based upon univariate analyses selecting variables with a *p* value < 0.1. The reciprocal of this probability was then used as a weight in the Cox regression model. Model diagnostics were performed to ensure that the assumptions of proportional hazards were not violated and to confirm that the weights were appropriately balanced without extreme weights or perfect prediction. No weights were greater than 16. Multivariable adjustments were made for age, gender, drug exposure and comorbidity. Analyses were undertaken using STATA version 13.1.

The vaccination survey was approved by both hospital clinical audit departments ements. Using the HRA decision tool and after seeking formal advice through the hospital Research and Development department, formal ethical approval was not required. All data collected was anonymised and retrospective and met local data protection requirements.

## Results

Baseline demographics of 929 patients included in analysis are presented in Table 1. Vaccination was primarily undertaken in the community setting (96 % of all vaccination) with pneumococcal and influenza vaccination rates similar across both sites. Influenza vaccination was offered to over 90% of patients(841/929)and was administered in 85.9% of patients (798/929) (Table 2). Pneumococcal vaccination rates were poor with 44 % of patients immunised.

**Table 1:** Baseline demographics population

	All *n* = 929	HES linked site only *n* = 387
Age (yrs.)	63.1	64.1
Gender Female n (%)	686 (74.9)	302 (78.1)
DMARDs n (%)	731 (78.7)	305 (78.8)
MTX n (%)	490 (52.7)	223 (57.6)
Biologics n (%)	240 (25.8)	109 (28.2)
Current smoker n (%)	191 (20.6)	54 (14.0)
Comorbidity n (%)	306 (32.9)	127 (32.8)

**Table 2:** Vaccination status of the study population

Total *n* = 929	Ever offered influenza vaccination	Ever offered pneumococcal vaccination	Received influenza vaccination	Received pneumococcal vaccination
*n* < 65 years = 467				
*n* > 65 years = 462				
Total n (%)	841 (90.5)	410 (44.1)	798 (85.9)	412 (44.3)
Age <65 years n (%)	421 (90.1)	203 (43.5)	400 (85.7)	207 (44.3)
Age >65 years n (%)	420 (90.9)	207 (44.8)	398 (86.1)	205 (44.4)

In patients not offered either vaccination, the main reason cited was that vaccination wasn’t recommended; 22 patients actively declined vaccination due to fear of worsening disease control. In a multivariable logistic regression model containing age, gender, smoking status, comorbidity and anti-rheumatic therapy, the only statistically significant variables to predict vaccination were comorbid illnesses (Odds Ratio [OR] 1.79 (95%CI 1.34 to 2.39)) and biologic therapy (OR 1.69 (95%CI 1.23 to 2.32)).

Overall 72/929 (7.8 %) patients reported at least 1 admission to secondary care with an infection in the preceding year. Limiting just to the hospital site with linkage to HES to validate reported infections, 35/327 (10.7 %) patients experienced at least 1 hospitalised infection. Three hundred and sixty-nine patients (40 %) reported being prescribed at least 1 antibiotic course by their primary care physician within the preceding year.

As the majority of patients were vaccinated against influenza, analysis of the effect of this vaccination on infection rate wasn’t possible. The rate of admission amongst patients who received pneumococcal vaccination was lower than those who reported not having received pneumococcal vaccination, although the odds of recalling an admission were not significantly different: OR 0.75 (95 % CI 0.46 to 1.24; *p* = 0.266). After adjusting for age, gender, comorbidity, and smoking status the OR was 0.61 (95 % CI 0.37 to 1.03).

## Discussion

This study is the largest UK survey of vaccination uptake in RA patients. The high uptake of influenza vaccination represents a positive and proactive attitude towards vaccination from both rheumatologists and primary care physicians. The findings of influenza vaccine uptake are very favourable with previously published audits and show successful uptake of vaccination in the community. There was a striking disparity between influenza and pneumococcal vaccine uptake. The reasons for this are likely to be multifactorial, including lack of awareness, organisation barriers to administration and possibly negative perception of the safety of vaccines [17].

Increasing public perception of both influenza and pneumococcal infection as significant threats to health and encouraging vaccination as an effective preventive strategy may correlate with a higher uptake of vaccination. Housden et al. reported a four-fold reduction in both admissions and case fatality rates due to respiratory tract infection in RA patients following the implementation of a successful ‘rheumatologist led’ vaccination programme [18]. Together with rationalising immunosuppressive agents and minimising steroid exposure, vaccination is a modifiable risk factor that can mitigate against infection. Although rheumatologists are responsible for the initiation of immunosuppressive agents, at present it remains in the domain of the GP to ensure vaccination administration. Primary care practices are commissioned to vaccinate people over 65 and those under 65 at risk (i.e. immunosuppressed patients) against influenza and pneumococcus and are remunerated accordingly through the NHS ‘Enhanced Services Payment’ scheme.

Fear of side effects of vaccination has previously been reported as a strong negative influence, while lack of general motivation and ignorance about the recommendations are other commonly reported barriers to both seasonal and pandemic vaccination [19]. Information pertaining to vaccination in RA is freely available to further educate patients on the risks of infection and benefits of vaccination; such resources should be utilised in routine clinical care (http://goo.gl/y1zvyp).

Biologic prescription was significantly associated with increased uptake of pneumococcal vaccination. This is likely to be a reflection of departmental protocols advising pre-screening of patients and written correspondence to GPs encouraging vaccine administration prior to the commencement of biologic therapy. Age was not a predictor of influenza or pneumococcal vaccination, a surprising observation considering the national schedule for vaccination advocating vaccination in people over 65. It was not possible to assess the impact of individual biologic agents on infection due to the relatively small sample size, however a recent review article has reviewed the current literature pertaining to individual DMARD and biologic agents and infection risk in RA [20].

Fewer infections were reported in patients who received pneumococcal vaccination. Although not a statistically significant finding, it is an intriguing observation as reported infections were not exclusively limited to pneumococcus. This may reflect bias within the study, or an alternative explanation is that the proportions of respiratory infections attributable to pneumococcus are underestimated.

Hospitalised infective episodes represent the tip of the iceberg when considering infections in RA. The infection rate in the study cohort was is in keeping with published rates and suggests that the study sample was representative of a standard RA population. It is advised that all patients should have a formal risk assessment of infection risk prior to commencing immunosuppressive agents. The ‘RABBIT’ tool (http://www.biologika-register.de/en/home/) helps to estimate the probability of serious infection within 12 months of commencing treatment and was developed with data from over 5000 patients with RA who were recruited to the German biologics registry with validation in a subsequent cohort of nearly 3000 patients [21, 22]. Although it doesn’t replace clinical judgement, the high agreement between observed and predicted infections allows clinicians to make informed decisions when balancing treatment versus infection risk. A particular strength of the RABBIT score is that it accommodates for time varying factors such as infections within previous 12 months. It is known that prior history of infection predicts future infection [21]. Caution must be exercised as the RABBIT score has not been validated in the UK RA patients.

Vaccine efficacy may also be affected by choice of immunosuppressive therapy. Methotrexate, rituximab and abatacept are associated with decreased immunogenicity to both influenza and pneumococcal vaccination, however TNF inhibitors, tocilizumab and sulfasalazine do not appear to have a negative effect on vaccine immunogenicity (albeit limited data for the latter two) [23]. Although the humoral response to vaccination may be muted with certain immunosuppressive agents, protective titres may still be achieved thus conferring the intended protective effect of vaccination. Reporting on 152 patients with RA, Coulson and colleagues found no significant correlation between pneumococcal antibody levels and time since pneumococcal vaccination and suggested that a single administration of pneumovax offered up to 10 years protection against the development of pneumococcal pneumonia in RA patients on methotrexate [24].

The nature of the survey study design means that it was prone to recall bias. Validation of vaccination status from GP and HES data was congruent with patient reported data. It is more likely that recall bias was associated with pneumococcal vaccination and the authors concede the true number of vaccinated subjects may have been underestimated. In future audits, correlation with the primary care record would ensure the data would be more robust with access to national HES data improving data capture of hospital admissions due to infection. Additionally, the number of antibiotic courses prescribed in the community was a self-reported variable and not confirmed with practice data and this is another source of bias. Comorbidity data may have been subject to misclassification bias, a perpetual risk of routinely captured data. There was likely selection bias in who was recruited to the audit as patients were not sent any reminders to complete the postal survey. It is difficult to predict the vaccination status of patients who chose not to participate in the survey.

A challenge in the analysis was addressing steroid exposure as it was not possible to account for intermittent steroid therapy, variable oral dosing over time or the administration of parenteral steroids. Thus no adjustment was made for the dose of steroid and its relationship to infection risk. The impact of steroid (even at modest doses of <7.5 mg daily) on infection risk is well established. Wolfe et al. demonstrated a dose related association between prednisolone dosage and pneumonia hospitalisation in patients with RA [25]. Steroid and infection risk in RA has been the subject of a meta-analysis of observational studies which found corticosteroids were associated with an increase in all-site serious infection (RR: 1.89;95 % CI: 1.60–2.24), lower respiratory tract infections (RR: 2.10; 95 % CI: 1.52–2.91), tuberculosis (RR: 1.74; 95 % CI: 1.09–2.76) and herpes zoster (RR: 1.74; 95 % CI: 1.28–2.36) with a dose-related increase in risk of infection [26].

It was not possible to correct for unmeasured cofounders such as severity of chronic lung disease which would impact on susceptibility to infection and choice of immunosuppressive agent. Prior hospitalisation is a predictor for future admissions with severe infection and it wasn’t possible to correct for this in the study but it is a factor that should be part of infection assessment in RA patients. It was not possible to demonstrate the relationship between pneumococcal vaccination and infection over time due to the size of the population, however this is an area of potential interest for future studies.

## Conclusions

This study highlights the need to raise the awareness of vaccination amongst patients and primary care providers in order to mitigate risk of infection. Pneumococcal vaccine uptake in particular consistently appears to be low. Pneumococcal and influenza vaccinations are safe in rheumatic disease and should be encouraged as part of holistic management in RA. Vaccination takes place primarily in the community setting, and pathways to improving vaccination will require engagement between primary and secondary care. The rheumatology community need to recognise this crucial link, acknowledging that our patients are at risk of falling between services if the rheumatologist does not take responsibility for ensuring vaccination uptake. In addition, rheumatologists should address modifiable risk factors, select the safest combinations of immunosuppression, and where possible minimise prescription of steroids. Regular assessment of infection risk and consideration of infection history are also key in ensuring holistic and personalized management of patients with RA.

## Abbreviations

BSR, British Society of Rheumatology; CI, confidence interval; HES, Hospital episode statistics; IPTW, inverse probability of treatment weighting; JCVI, Joint Committee on Vaccination and Immunisation; KCH, King’s College Hospital, London; OR, odds ratio; RA, rheumatoid arthritis; SMR, standardised mortality ratio

## Additional file

Additional file 1: Vaccination questionnaire. (DOCX 184 kb)

## References

[1] Scott DL, Wolfe F, Huizinga TWJ (2010). Rheumatoid arthritis. Lancet.

[2] Young A, Koduri G, Batley M, Kulinskaya E, Gough A, Norton S, Dixey J, Early Rheumatoid Arthritis Study (ERAS) group (2007). Mortality in rheumatoid arthritis. Increased in the early course of disease, in ischaemic heart disease and in pulmonary fibrosis. Rheumatol.

[3] Doran MF, Crowson CS, Pond GR, O’Fallon WM, Gabriel SE (2002). Frequency of infection in patients with rheumatoid arthritis compared with controls: a population-based study. Arthritis Rheum.

[4] Smitten AL, Choi HK, Hochberg MC, Suissa S, Simon TA, Testa MA, Chan KA (2008). The risk of hospitalized infection in patients with rheumatoid arthritis. J Rheumatol.

[5] Franklin J, Lunt M, Bunn D, Symmons D, Silman A (2007). Risk and predictors of infection leading to hospitalisation in a large primary-care-derived cohort of patients with inflammatory polyarthritis. Ann Rheum Dis.

[6] Starkebaum G (2002). Chronic neutropenia associated with autoimmune disease. Semin Hematol.

[7] Wagner UG, Koetz K, Weyand CM, Goronzy JJ (1998). Perturbation of the T cell repertoire in rheumatoid arthritis. Proc Natl Acad Sci U S A.

[8] Koetz K, Bryl E, Spickschen K, O’Fallon WM, Goronzy JJ, Weyand CM (2000). T cell homeostasis in patients with rheumatoid arthritis. Proc Natl Acad Sci U S A.

[9] Au K, Reed G, Curtis JR, Kremer JM, Greenberg JD, Strand V, Furst DE, CORRONA Investigators (2011). High disease activity is associated with an increased risk of infection in patients with rheumatoid arthritis. Ann Rheum Dis.

[10] Dixon WG, Watson K, Lunt M, Hyrich KL, Silmanm AJ, Symmons DPM (2006). Rates of serious infection, including site-specific and bacterial intracellular infection, in rheumatoid arthritis patients receiving anti-tumor necrosis factor therapy: Results from the British Society for Rheumatology Biologics Register. Arthritis Rheum.

[11] Thomas E, Symmons DPM, Brewster DH, Black RJ, Macfarlane GJ (2003). National study of cause-specific mortality in rheumatoid arthritis, juvenile chronic arthritis, and other rheumatic conditions: A 20 year followup study. J Rheumatol.

[12] Lim WS, Macfarlane JT, Boswell TC, Harrison TG, Rose D, Leinonen M, Saikku P (2001). Study of community acquired pneumonia aetiology (SCAPA) in adults admitted to hospital: implications for management guidelines. Thorax.

[13] Bewick T, Sheppard C, Greenwood S, Slack M, Trotter C, George R, Lim WS (2012). Serotype prevalence in adults hospitalised with pneumococcal non-invasive community-acquired pneumonia. Thorax.

[14] Sowden E, Mitchell WS (2007). An audit of influenza and pneumococcal vaccination in rheumatology outpatients. BMC Musculoskelet Disord.

[15] Haroon M, Adeeb F, Eltahir A, Harney S (2011). The uptake of influenza and pneumococcal vaccination among immunocompromised patients attending rheumatology outpatient clinics. Joint Bone Spine.

[16] Thomas LM, Wood A, Shenton S, Ledingham JM (2005). Uptake of influenza vaccination in rheumatology patients: reply. Rheumatology.

[17] Findlay PF, Gibbons YM, Primrose WR, Ellis G, Downie G (2000). Influenza and pneumococcal vaccination: patient perceptions. Postgrad Med J.

[18] Housden MM, Bell G, Heycock CR, Hamilton J, Saravanan V, Kelly CA (2010). How o reduce morbidity and mortality fro chest infections in rheumatoid arthritis. Clin Med.

[19] Dexter LJ, Teare MD, Dexter M, Siriwardena AN, Read RC (2012). Strategies to increase influenza vaccination rates: outcomes of a nationwide cross-sectional survey of UK general practice. BMJ Open.

[20] Subesinghe S, Whittaker M, Galloway J (2015). Mitigating infection risk with immunotherapy in rheumatoid arthritis. Int J Clin Rheum.

[21] Strangfeld A, Eveslage M, Schneider M, Bergerhausen HJ, Klopsch T, Zink A, Listing J (2011). Treatment benefit or survival of the fittest: what drives the time-dependent decrease in serious infection rates under TNF inhibition and what does this imply for the individual patient?. Ann Rheum Dis.

[22] Zink A, Manger B, Kaufmann J, Eisterhues C, Krause A, Listing J, Strangfeld A (2014). Evaluation of the RABBIT Risk Score for serious infections. Ann Rheum Dis.

[23] Whittaker M, Galloway J, Subesinghe S (2014). Systematic review of the effect of anti-rheumatic therapies upon vaccine immunogenicity. Arthritis Rheum.

[24] Coulson E, Saravanan V, Hamilton J, Long KS, Morgan L, Heycock C, Kelly C (2011). Pneumococcal antibody levels after pneumovax in patients with rheumatoid arthritis. Ann Rheum Dis.

[25] Wolfe F, Caplan L, Michaud K (2006). Treatment for rheumatoid arthritis and the risk of hospitalization for pneumonia. Ann Rheum Dis.

[26] Dixon WG, Abrahamowicz M, Beauchamp M-E, Ray DW, Bernatsky S, Suissa S, Sylvestre M-P (2012). Immediate and delayed impact of oral glucocorticoid therapy on risk of serious infection in older patients with rheumatoid arthritis: a nested case–control analysis. Ann Rheum Dis.

