# Supplementary material for: A large two-centre study in to rates of influenza and pneumococcal vaccination and infection burden in rheumatoid arthritis in the UK
Source: BMC Musculoskelet Disord. 2016 Aug 4;17:322. doi: 10.1186/s12891-016-1187-4 (PMC4973522; doi:10.1186/s12891-016-1187-4)
Supplement: Additional file 1: — Vaccination questionnaire. (DOCX 184 kb) [file 12891_2016_1187_MOESM1_ESM.docx]

**A survey of vaccination in patients with rheumatic disease**

Thank you for taking the time to help us with this survey.

As you may be aware, people with rheumatic conditions can be more susceptible to infections (e.g. flu and chest infections). As a result, there is a national recommendation that we offer people with rheumatic diseases the opportunity to be immunised against such infections. Vaccinations can help reduce the risk of infection and have been shown to be safe in people with rheumatic diseases. These vaccinations are administered in primary care from GP surgeries and include the Influenza (on a yearly basis) and a one off pneumococcal vaccinations. We are currently evaluating how many of the patients under our care receive these vaccinations.

We would be grateful if you would complete the attached questionnaire (which should take approximately 3 - 5 minutes) about your recent vaccinations and return it to the vaccination response box located in Suite 3. If you have already completed this survey via post there is no need to repeat the survey. The results of the study will help us improve our practice and ensure we are providing the best possible care to you.

Thank you for your participation.

Dr Sujith Subesinghe – Rheumatology Registrar, King’s College Hospital, London.

Dr James Galloway – Consultant Rheumatologist, King’s College Hospital, London

| VACCINATION QUESTIONNAIRE | | | | | |
| --- | --- | --- | --- | --- | --- |
| This questionnaire will provide valuable information regarding your rheumatological condition and your vaccination history. | | | | | |
| Patient Hospital Number: | |  | | | |
| Patient NHS Number:  *(if known)* | |  | | | |
| Date of Birth: (DD/MM/YY) | |  | | | |
| GENERAL QUESTIONS ABOUT YOU: | | | | | |
| 1. | What is your rheumatology diagnosis?  (tick one) | |  | Rheumatoid arthritis | |
|  |  |  |  | Psoriatic arthritis | |
|  |  |  |  | Ankylosing spondylitis | |
|  |  |  |  | Systemic lupus erythematosus | |
|  |  |  |  | Connective tissue disease (not SLE) | |
|  |  |  |  | Vasculitis | |
|  |  |  |  | Other (please specify) | |
| 2. | In which year were you diagnosed? | | .................................................. | | |
| 3. | Which of the following medications are you currently taking for your rheumatology condition?  (tick all that apply) | |  | Prednisolone | Dosage: ....................... |
|  |  |  |  | Methotrexate | |
|  |  |  |  | Sulphasalazine | |
|  |  |  |  | Hydroxychloroquine | |
|  |  |  |  | Leflunomide | |
|  |  |  |  | Mycophenolate | |
|  |  |  |  | Azathioprine | |
|  |  |  |  | Anti-TNF injections Enbrel/Humira/Remicade/Cimzia/Simponi | |
|  |  |  |  | Rituximab (Mabthera) | |
|  |  |  |  | Tocilizumab (Ro-Actemra) | |
|  |  |  |  | Abatacept (Orencia) | |
|  |  |  |  | Other DMARD (please specify) | |
| 4. | Have you ever been diagnosed with any of the following conditions?  (tick all that apply) | |  | Diabetes | |
|  |  |  |  | Coronary heart disease (angina, heart attack) | |
|  |  |  |  | Stroke/TIA | |
|  |  |  |  | Chronic lung disease (including asthma and chronic bronchitis) | |
|  |  |  |  | Chronic kidney disease | |
|  |  |  |  | Chronic liver disease | |
| VACCINATION SPECIFIC QUESTIONS: | | | | | |
| 5. | Have you been offered any of the following vaccinations?  (tick as appropriate) | |  | Influenza | |
|  |  |  |  | Pneumonia | |
|  |  |  |  | Shingles | |
|  |  |  |  | Hepatitis | |
| 6. | Have you ever had a vaccination against influenza? | | Yes  No  if Yes please answer 6.a. and b. | | |
| 6.a | Date of last influenza vaccination *(month and year)*? | | .................................................. | | |
| 6.b. | Where did you receive your influenza vaccination? | | | | |
| i. | In hospital | |  | | |
| ii. | At your GP surgery | |  | | |
| iii. | At work | |  | | |
| iv. | Elsewhere | | If ticked; please specify in the box below | | |
| iv. |  | | | | |
| 7. | Have you ever had a vaccination against pneumonia? | | Yes  No  if Yes please answer 7.a. and b. | | |
| 7.a | Date of last pneumonia vaccination *(month and year)*? | | .................................................. | | |
| 7.b. | Where did you receive your pneumonia vaccination? | | | | |
| i. | In hospital | |  | | |
| ii. | At your GP surgery | |  | | |
| iii. | At work | |  | | |
| iv. | Elsewhere | | If ticked; please specify in the box below | | |
| iv. |  | | | | |
| 8. | If you have not received any vaccinations in the last 5 years what was the reason for this? (tick all that apply) | | | | |
| 8.a. | I was not aware that it was recommended for me | |  | | |
| 8.b. | I have never been offered any vaccinations | |  | | |
| 8.c. | I declined vaccination | |  | | |
| 8.d. | Other | | (If yes; please specify why in the box below) | | |
| 8.d. |  | | | | |
| 9. | Have you had an infection in the last year (January 2013- present) that was severe enough that you had to go to hospital for treatment? | | Yes  No | | |
| 10. | If you have been admitted to hospital because of an infection, how many times has this happened in the past year (January 2013-present)? | | .................................................. | | |
| 11. | Has your GP prescribed you oral antibiotics for an infection in the past year (January 2013-present)? | | Yes  No | | |
| 12. | How many times in the past year (January 2013-present) have you had an infection for which your GP prescribed you antibiotics? | | .................................................. | | |
| 13. | What is your current smoking status? | |  | Never smoked | |
|  |  |  |  | Current smoker | |
|  |  |  |  | Ex-smoker | |
| 14. | Further information: Please provide any further comments about vaccinations and infections | | | | |
|  |  | | | | |

THANK YOU

Thank you for taking the time to complete this questionnaire

Please return the questionnaire to the Clinic Nurse
